# Low back pain in older adults: risk factors, management options and future directions

**DOI:** 10.1186/s13013-017-0121-3

**Published:** 2017-04-18

**Authors:** Arnold YL Wong, Jaro Karppinen, Dino Samartzis

**Affiliations:** 1 Department of Rehabilitation Sciences, Faculty of Health and Social Sciences, The Hong Kong Polytechnic University, Hung Hom, Hong Kong, SAR China; 2 Medical Research Center Oulu, Department of Physical and Rehabilitation Medicine, University of Oulu and Oulu University Hospital, Oulu, Finland; 3 Finnish Institute of Occupational Health, Oulu, Finland; 4 Department of Orthopaedics and Traumatology, The University of Hong Kong, Pokfulam, Hong Kong, SAR China

**Keywords:** Risk factors, Spine, Disc degeneration, Management, Low back pain, Elderly, Genetics, Falls, Brain, Pain assessment

## Abstract

Low back pain (LBP) is one of the major disabling health conditions among older adults aged 60 years or older. While most causes of LBP among older adults are non-specific and self-limiting, seniors are prone to develop certain LBP pathologies and/or chronic LBP given their age-related physical and psychosocial changes. Unfortunately, no review has previously summarized/discussed various factors that may affect the effective LBP management among older adults. Accordingly, the objectives of the current narrative review were to comprehensively summarize common causes and risk factors (modifiable and non-modifiable) of developing severe/chronic LBP in older adults, to highlight specific issues in assessing and treating seniors with LBP, and to discuss future research directions. Existing evidence suggests that prevalence rates of severe and chronic LBP increase with older age. As compared to working-age adults, older adults are more likely to develop certain LBP pathologies (e.g., osteoporotic vertebral fractures, tumors, spinal infection, and lumbar spinal stenosis). Importantly, various age-related physical, psychological, and mental changes (e.g., spinal degeneration, comorbidities, physical inactivity, age-related changes in central pain processing, and dementia), as well as multiple risk factors (e.g., genetic, gender, and ethnicity), may affect the prognosis and management of LBP in older adults. Collectively, by understanding the impacts of various factors on the assessment and treatment of older adults with LBP, both clinicians and researchers can work toward the direction of more cost-effective and personalized LBP management for older people.

## Background

The average lifespan of humans has dramatically increased in the last decade due to the advance in medicine [1]. According to the United Nations, the world population of individuals aged 60 years or above will triple by 2050 [2]. In the UK alone, approximately 22% of the population will be 65 years or older by 2031, exceeding the number of those aged less than 25 years [3]. However, the fast-growing aging population also increases the likelihood of non-communicable diseases (e.g., musculoskeletal complaints). Studies have suggested that the prevalence of musculoskeletal pain in older adults ranges from 65 to 85% [4, 5], with 36 to 70% of them suffering from back pain [5, 6].

Low back pain (LBP) is the most common health problem among older adults that results in pain and disability [4, 7–10]. Older adults, aged 65 years or above, are the second most common age group to visit physicians for LBP [11]. Earlier research suggests that LBP prevalence progressively increases from teenage [12] to 60 years of age and then declines [13–16], which may be ascribed to occupational exposure among working-age adults [17, 18], or age-related changes in pain perception or stoicism [19]. However, recent studies have revealed that LBP remains ubiquitous among older adults at their retirement ages [20, 21]. In population-based studies, the 1-year prevalence of LBP in community-dwelling seniors ranged from 13 to 50% across the world [4, 13, 22–24]. Similarly, while up to 80% of older residents in long-term care facility experience substantial musculoskeletal pain [25–27] and one-third of these cases are LBP [28], often older residents’ pain is underreported and inadequately treated [25–27].

It is noteworthy that both the incidence and prevalence of severe and chronic LBP increase with older age [13, 29, 30]. Docking et al. [17] reported that the 1-month prevalence of disabling back pain (pain that affected daily activities within the past month) increased from 3.8% among people aged between 77 and 79 years to 9.7% among those aged between 90 and 100 years. Williams and coworkers [31] also found that individuals aged 80 years or above were three times more likely to experience severe LBP than those aged between 50 and 59 years. Because severe LBP usually results in poor treatment outcomes and functional disability [17, 32], timely LBP management of older adults is crucial. Importantly, compared to working-age adults, older adults aged 65 years or above are more likely to develop chronic LBP that lasts for more than 3 months [13, 33]. A Spanish study found that the prevalence rates of chronic LBP among females and males aged 65 years or older were 24.2 and 12.3%, respectively [34], while an Israeli study documented that the prevalence of chronic LBP in people aged 77 years was as high as 58% [35].

Notwithstanding the high prevalence of LBP among older adults, their pain is usually undertreated. A recent study showed approximately 25% of senior nursing home residents with chronic pain did not receive analgesics, and only 50% of all analgesics were prescribed as standing orders at suboptimal doses, which did not follow geriatric clinical guidelines [36, 37]. According to those guidelines, older patients with chronic pain should receive analgesics as a standing dose rather than on an as-needed basis in order to ensure adequate concentration of analgesic in serum for continuous pain relief [36, 38]. Standing-dose analgesics are particularly important for people with cognitive impairment because they cannot appropriately request medication.

While undertreatment of LBP in older adults may be ascribed to the avoidance of high-dose analgesics (e.g., opioid) prescription, it may also be attributed to the difficulty in identifying the presence or causes of LBP. Research has shown that less than 50% of primary care physicians have strong confidence in diagnosing the causes of chronic LBP in older adults [32]. Consequently, this may result in over-reliance on medical imaging or improper LBP management (e.g., undertreatment). Imperatively, untreating or undertreating older adults with LBP may result in sleep disturbances, withdrawal from social and recreational activities, psychological distress, impeded cognition, malnutrition, rapid deterioration of functional ability, and falls [39]. These LBP-related consequences may compromise their quality of life and increase their long-term health care expenses [40].

Although various medical associations have published clinical guidelines on conservative management of chronic pain in older adults [37, 41, 42], there is paucity of literature summarizing various causes or risk factors of developing severe/chronic LBP among older adults. Since a better understanding of these factors can improve LBP management, the objectives of the current narrative review were to summarize potential causes of LBP, risk factors for chronic LBP, special consideration for LBP management (e.g., pain evaluations among patients with dementia) in older people aged 60 years or older, and future research directions.

### Search strategies and selection criteria

Potential articles were identified for review through PubMed from January 1, 1990, to November 30, 2016. Search terms included keywords and medical subject headings related to “low back pain,” “LBP,” “older adult*,” “senior*,” “elderly,” “cognitive impairment,” “dementia,” “nonverbal,” “community-dwelling,” “nursing home,” “long-term care facilities,” “risk factor*,” “brain,” “genetics,” “assessment*,” and “intervention*.” Various Boolean terms were used in conjunction with various search terms. Articles were selected based on the relevance of topic and restricted to the English language. The reference lists of relevant articles were also included for review. A total of 2182 citations were identified from the search. Of them, information from 320 articles was used in the current review.

### Potential causes of low back pain

#### Non-specific or mechanical low back pain

Like among young adults, the majority of LBP among older adults has no definite pathology (e.g., fracture or inflammation) and is diagnosed as non-specific LBP. These patients experience LBP that is altered by posture, activity, or time of the day. Non-specific LBP may originate from different pain sources [43]. Disc degeneration on magnetic resonance imaging (MRI) is more prevalent with age progression and as such in older adults; however, it is less likely to be the pain source as compared to young adults [44]. Conversely, facet joint pain in seniors may present as localized LBP with or without posterior thigh pain during walking. The pain may be aggravated during trunk extension, ipsilateral lateral flexion, and/or rotation [45]. Lumbar degenerative spondylolisthesis (defined as forward or backward slippage of a cephalic vertebra over a caudal one secondary to a degenerated disc and altered facet joint alignment) is common among women aged 60 years or older and is usually associated with facet hypertrophy [46]. The presence of degenerative spondylolisthesis alongside facet hypertrophy and thickening of ligamentum flavum may results in pain, spinal stenosis, and neurological deficits in older adults [46, 47]. Although spinal degenerative changes may induce LBP, not all anomalies on lumbar medical imaging are related to LBP because abnormal imaging phenotypes are ubiquitous among asymptomatic older adults [44, 48–50].

Additionally, non-specific LBP may originate from structures other than the lumbar spine. Many older patients with chronic LBP display physical findings comparable to sacroiliac joint pain (83.6%) and myofascial pain (95.5%) [51]. Symptoms of sacroiliac joint disorders are similar to facet joint pain, which includes localized LBP with or without posterior thigh pain that can be alleviated by lying [52]. Myofascial pain is a localized palpable tenderness and tightness within a muscle that resists passive stretching and reproduces predictable referred pain pattern on palpation [53]. Myofascial pain in lumbar muscles or piriformis are common among seniors. Collectively, it is difficult to identify the sources of non-specific LBP because its causes are usually multifactorial. Various factors (e.g., anxiety, depression, coping strategies, and pain genes) can modify the severity and chronicity of LBP [31, 35, 50].

#### Radiculopathy

While non-specific LBP is usually localized at the lumbar region and/or thigh, the compression of nerve roots or spinal meninges by degenerated spinal structures (e.g., herniated discs, facet joints, and/or epidural fat) [54] may lead to radiculopathy that radiates distal to the knee. The clinical presentation of radiculopathy depends on the location of neural tissue compression. Lumbar spinal stenosis (LSS) secondary to degenerative changes (e.g., osteophytes and hypertrophic ligamentum flavum) at a single or multiple level(s) may lead to unilateral or bilateral radiculopathy and neurogenic claudication with or without LBP [55–57]. Neurogenic claudication is characterized by numbness and heaviness of legs after prolonged walking, which can be eased by a flexed position (e.g., forward leaning or sitting) [58–60]. On the contrary, the presence of osteophyte/narrowing in the lateral recess or in the vertebral foramen may result in radicular leg pain without LBP [61]. Research on asymptomatic or some clinical populations have suggested that the prevalence of degenerative LSS ranged from 6 to 13.1% [62, 63] and the rate increases with age [64]. A population-based imaging study found that the prevalence of degenerative LSS (i.e., ≤10-mm anteroposterior diameter of spinal canal) in young (<40 years) and older adults (>60 years) were 4.0 and 14.3%, respectively [64].

#### Osteoporotic vertebral fractures

Given the hormonal changes following menopause, women are more susceptible to osteoporotic fracture and related LBP [65, 66]. Approximately 25% of all postmenopausal women suffer from vertebral compression fracture and the prevalence of this condition increases with age [65]. It is estimated that the prevalence of vertebral compression fracture in women aged 80 years or above can be as high as 40% [65]. As compared to patients with non-specific LBP, patients with vertebral fractures experience more disability [67]. Unfortunately, only one third of the cases are correctly diagnosed because many seniors assume bone and joint pain as part of the aging process [68]. As such, physicians should pay more attention to examine seniors with acute onset of localized LBP that may or may not present with paraspinal muscle spasm. A recent systematic review suggests that older age, corticosteroid use, and significant trauma are the risk factors for vertebral fractures [69]. The common site of compression fractures occur at the thoracolumbar region [70–72]. Depending on the mechanism of fractures, some vertebral compression fracture may result in radiculopathy. The most common fracture mechanism is due to a flexion movement or trauma that causes an anterior wedge fracture [73]. Since the posterior vertebral body remains intact and the collapsed anterior vertebra heals without regaining height, it will result in a kyphotic deformity without compromising the spinal cord [73]. Another type of vertebral compression fracture involves the center part of the vertebral body without affecting the anterior or posterior wall. This type of fracture does not affect the spinal cord. A less common osteoporotic vertebral fracture involves the axial compression of the entire vertebral body or the posterior portions of the vertebra that may compress the spinal canal and results in neurological deficit [71–73].

#### De novo degenerative lumbar scoliosis

De novo degenerative lumbar scoliosis (DNDLS) is a spinal deformity in older adults that results in disabling LBP/leg pain and suboptimal quality of life. [74–76]. DNDLS is defined as a lumbar scoliotic curve with a Cobb angle ≥10° in the coronal plane that develops after 50 years of age in people without a history of adolescent idiopathic scoliosis. [77]. The reported prevalence of DNDLS in the adult population has ranged from 8.3 to 13.3% [78–80], while that in adults older than 60 years was as high as 68% [81]. Multifactorial causes have been suggested for DNDLS, including intervertebral disc degeneration and genetic predisposition [82–84]. It is believed that the asymmetrical biomechanical load on the vertebral endplate on the concave side of the curve may cause inflammatory responses in the endplate and adjacent bone marrow of the vertebral body, which may result in LBP. [85–87]. This premise has been substantiated by a recent study that found (1) bone marrow edema in DNDLS was more prevalent in older adults with LBP than those without LBP, (2) bone marrow edema was more frequent on the concave side of the DNDLS curve, and (3) the location of bone marrow edema on MRI was closely associated with local lumbar tenderness [87]. However, no significant relation between Cobb angle and LBP symptoms in older adults has been reported [81]. Interestingly, the curve progression rate of DNDLS is higher than that of adolescent idiopathic scoliosis [77]. Three radiological variables (i.e., increased intervertebral disc degeneration, an intercrest line passing through the L5 level (not L4 or higher), and apical lateral vertebral translation for at least 6 mm)) have been identified as predictors of DNDLS curve progression [77].

#### Tumors/cancers

The incidence rates for all neoplasms exponentially increase with age [88] although only less than 1% of the causes of LBP presented to primary care physicians are attributed to spinal tumors [89]. A majority of these tumors are related to metastasis and only a handful of them are primary tumors [90–95]. The common metastatic sources of LBP are prostate and kidney although primary malignant tumors (e.g., chordoma, plasmacytoma, or lymphoma) are also be found in older adults [90]. Unlike young adults, seniors are unlikely to have primary benign tumors (e.g., osteoblastoma, osteochondroma, osteoma, eosinophilic granuloma, and aneurysmal bone cysts). Clinically, typical symptom of spinal tumors is progressive, unremitting, localized, or radiating pain that are aggravated by movement, worse at night, and cannot be eased by rest. In addition, patients may experience weakness and feel the presence of a lump [96].

#### Spinal infection

Vertebral osteomyelitis (VO) is a life-threatening infectious musculoskeletal disease in older people caused by an infection of vertebral bones [97]. Given the growing aging population, the incidence of VO is increasing [98–100]. Although the reported incidence rate of VO in the general population only ranges from 2.5 cases to 7 cases per 100,000 people-years [99, 101], the mortality of these patients can be as high as 12% [99, 102]. Four causes of VO have been suggested. First, pathogenic bacteria may be disseminated hematogenously from a distant infected source and multiply at the metaphyseal arterioles of vertebral bone that causes microabscess formation, bone necrosis, and fistula within bone [103]. *Staphylococcus aureus* is the most common pathogen. Second, tubercular VO may occur in seniors who have contracted tuberculous infection at young age. *Mycobacterium tuberculosis* may be transmitted to and remains in the vertebral bone. Age-related deterioration of the host’s immunity or certain incidences (e.g., osteoporosis, trauma, or non-myobacterial infections) may reactivate *M. tuberculosis* in the bone that causes osteomyelitis. Third, aerobic gram-negative bacilli in older men with urinary tract infection may rarely reach the lumbar spine through Batson’s plexus and cause VO [97]. Fourth, iatrogenic infection following spinal surgeries or injections may cause vertebral osteomyelitis. Clinically, patients with VO may present with fever, elevated C-reactive protein, paraspinal muscle spasm, LBP, neurological deficits, and epidural abscess. Additionally, patients with tuberculous osteomyelitis may have a groin mass because of the presence of abscess in psoas muscle [97]. Taken together, greater age and certain comorbidities (e.g., diabetes, hemodialysis usage, liver cirrhosis, malignancy, and infectious endocarditis) are known to increase inpatient mortality of VO [99]. Clinicians should be suspicious of VO if older patients with the abovementioned comorbidities demonstrate unidentified fever and/or LBP [99]. Clinical findings, laboratory results, bone scintigraphy, and/or spinal biopsy are usually used to make differential diagnosis of VO.

Similarly, older people are more prone to develop pyogenic spondylodiscitis, which involves the infection of disc and adjacent vertebral bones. It has been estimated that the incidence rate of non-tuberculous or non-postoperative spondylodiscitis in the general population is approximately 0.2 to 2.4 cases per 100,000 people-years [101, 104–106], while that for people over 65 years old is as high as 9.8 cases per 100,000 person-years [107]. A recent population-based study reported that males aged 70 years or older displayed six times higher incidence rate of pyogenic non-tuberculosis spondylodiscitis than males under 70 years old. Likewise, females aged 70 years or above were three times more likely to exhibit pyogenic non-tuberculosis spondylodiscitis than younger counterparts [98]. Clinical presentations of spondylodiscitis are comparable VO. *S. aureus* is the major cause of pyogenic spondylodiscitis [108], while other bacteria (e.g., Streptococcus and Pneumococcus) may also cause the disease [98]. Magnetic resonance imaging is the gold standard for imaging pyogenic spondylodiscitis, which is visualized as reduced signal intensity of the affected disc and adjacent vertebral bodies with unclear endplates definition on T1-weighted images and enhanced signal intensity on T2-weighted images [109].

#### Visceral diseases

Since it is not uncommon for seniors to have co-morbidities, it is important to consider other non-spinal pathologies that usually present as chronic LBP. Several visceral diseases (e.g., dissecting abdominal aortic aneurysm, cholecystolithiasis, nephrolithiasis, prostatitis, urinary tract infection, and pelvic inflammatory disease) have known to generate symptoms comparable to chronic LBP [110].

#### Cauda equina syndrome

This syndrome is ascribed to the compression of multiple lumbar and sacral nerve roots in the spinal canal that lead to bowel, bladder, and/or sexual dysfunction, as well as perianal region numbness [111]. Depending on the location of nerve roots compression, patients with cauda equina syndrome may or may not experience sciatica. Potential causes of this syndrome include central disc herniation or spondylolisthesis at the lower lumber levels, spinal tumors, dislocated fracture, and abscess within the spinal canals [111]. Additionally, this syndrome may be secondary to some rare iatrogenic causes (e.g., spinal anesthesia or postoperative hematoma).

### Risk factors of developing severe/chronic low back pain in older adults

**Fig. 1:**
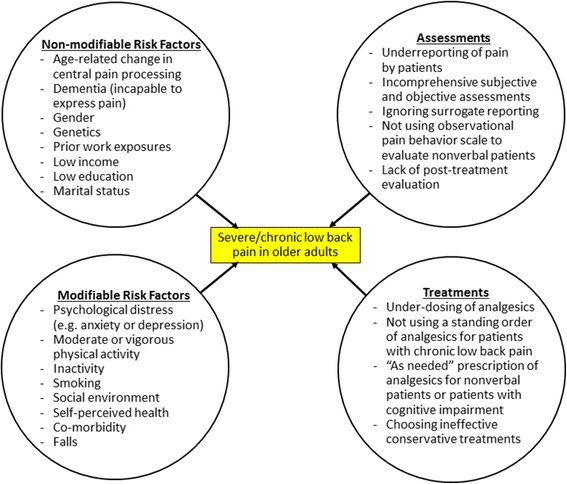
Factors affecting the development of severe or chronic low back pain among older adults

Although most LBP is self-limiting and begins to improve after a few days and resolves within a month [110], some patients are susceptible to chronic LBP that lead to significant disability. While age is a well-known risk factor for chronic LBP [112], other factors may perpetuate LBP in older adults (Fig. 1). The understanding of these factors can help identify high-risk patients and improve their LBP management. Since older adults usually face both age-related physical and psychosocial issues, comprehensive assessments and treatments are needed to effectively manage LBP in seniors.

### Non-modifiable risk factors

#### Altered supraspinal pain processing

Recent evidence suggests that normal aging may be associated with alterations in pain perception [113, 114] central pain processing [114] and/or neuroplastic changes to pain responses [115]. Both experimental pain and functional neuroimaging studies have found that older people display age-related increase in the heat pain threshold [116] and reduced responses in middle insular and primary somatosensory cortices toward a 44 °C heat stimulus [117]. These age-related neuropsychological changes in pain processing may reduce older peoples’ awareness and reporting of pain that may lead to undiagnosed health problems/injuries.

Conversely, some psychophysical studies reported that older adults displayed lower tolerance to various types of pain stimuli (e.g., ischaemic, mechanical, electrical, heat, or cold) [113, 114, 118] decreased pain thresholds for mechanical pressure [114, 116] or ischemic pain stimuli [119] and higher pain rating for noxious stimuli as compared to young adults [120]. Although speculative, the increased pain sensitivity in older adults may be attributed to diminished descending pain inhibition in older adults. Neuroimaging studies have shown that the volumes of brain regions responsible for pain processing (i.e., the cingulate, insula, striatum, hippocampus, cerebellum, and prefrontal cortex) significantly reduce as people age [121–126]. These findings may indicate age-related reduction in perceptual motor processing, diminished coordination of inhibitory motor response to noxious stimuli, and/or impaired descending endogenous pain inhibitory modulation [127–130]. Since patients with fibromyalgia are known to have significantly less striatal release of dopamine in response to experimental muscle pain [131] and people with chronic LBP are characterized by regional decreases in gray matter density in bilateral striatum (especially nucleus accumbens, putamen, and caudate) [132], the reduced pain-related striatal activity in seniors may indicate age-related impairment in endogenous pain modulation [127–129].

Additionally, age-related changes in neuroplasticity may decrease the pain tolerance in older adults. Compared to younger individuals, older people tend to show more rapid temporal summation of noxious heat stimuli in their central nervous system [116, 133–135]. Similarly, older adults display a prolonged period of capsaicin-induced hyperalgesia that may lead to relentless pain sensitization and sluggish resolution of neuroplastic change [115]. Importantly, the central pain processing can be further complicated by dementia-related neurodegeneration [113, 136]. Depending on the severity, locations or types of neurodegenerative changes, seniors with dementia or Alzheimer’s disease have demonstrated increased pain threshold and tolerance [137] or decreased pain threshold [138, 139]/pain tolerance [140]. Taken together, age-related changes in central pain processing of older adults may contribute to severe or chronic LBP in seniors.

Importantly, people with chronic back pain suffer from global and regional changes in functional connectivity and/or gray matter density in the brain that may perpetuate persistent pain [132, 141]. Human resting-state functional MRI research has revealed that, as compared to asymptomatic individuals, patients with chronic pain (i.e., back pain, osteoarthritis, and complex pain regional syndrome) demonstrate significantly decreased functional connectivity of the whole-brain and diminished regional connectivity in specific brain regions (e.g., supplementary motor cortex, mid-anterior cingulate cortex, superior parietal lobe, and part of the somatosensory network) but enhanced connectivity in thalamus and hippocampus [141]. These patients also display changes in allegiance of insula nodes or some lateral parietal nodes to certain brain modules (e.g., the sensorimotor brain module, default–mode network module, and attention module) [141]. These findings indicate that chronic pain is associated with decreased motor planning (supplementary motor cortex) and attention (superior parietal lobe) but increased somatosensory inputs to the cortex (thalamus) and chronification (hippocampus) [142, 143]. Similarly, a 1-year longitudinal study showed that people who experienced persistent back pain during the study period demonstrated significant decreases in global gray matter density as compared to healthy controls and patients who recovered during the period [132]. The same study found that patients with persistent back pain had significant regional decreases in gray matter density at bilateral nucleus accumbens (a key mesolimbic region), insula (pain perception cortex) [144–146], and left primary sensorimotor cortex, yet reduced negative functional connectivity between insula and precuneus/dorsolateral prefrontal cortex, and diminished functional connectivity of primary sensorimotor cortex [132]. The consistent findings of various studies suggest that chronic pain may lead to global and/or regional disruption of functional connectivity and structures of the brain that may hinder the treatment effectiveness for people with a history of recurrent or chronic pain [141].

#### Gender

Females are more susceptible to chronic LBP than males regardless of age [20, 31, 34, 112]. Jimenez-Sanchez and coworkers [34] estimated that women were two times more likely to develop chronic LBP than men. The higher prevalence of chronic pain in females may be attributed to complex biopsychosocial mechanisms (e.g., less efficient pain, habituation or diffuse noxious inhibitory control [147], genetic sensitivity, pain coping [148], and a higher vulnerability to develop temporal summation of chemically [149] or mechanically evoked pain) [150]. Further, women commonly have a higher number of concomitant chronic diseases (e.g., osteoporosis, osteopenia, and osteoarthritis), which are known to be risk factors for developing chronic LBP and psychological distress in older adults [34, 112].

#### Genetic influences

Recent research has highlighted that genetic factors play an imperative role in modulating pain sensitivity, responses to analgesics, and vulnerability to chronic pain development [50]. Some genetic factors not only predispose people to spinal disorders (e.g., scoliosis [151] and intervertebral disc degeneration [152, 153]) but also alter brain structures [154, 155] that may modify central pain processing and perception [156]. For instance, polymorphisms of the catechol-O-methyltransferase gene are known to affect the cognitive and emotion processing of pain in the brain [156]. While variations in some gene expression (e.g., val^158^met single-nucleotide polymorphism (SNP)) may modulate temporal summation of pain [157], other SNPs (e.g., catechol-O-methyltransferase gene, interleukin-6 GGGA haplotype or SCN9A gene, or hereditary sensory neuropathy type II gene) may alter pain sensitivity through different mechanisms (e.g., affecting voltage-gated sodium channels, altering myelination of nerve fibers, or modulating anabolism/catabolism of catecholamine neurotransmitters) [158–163]. Collectively, some people (including seniors) may be more susceptible to develop chronic LBP because of their genetic makeup. Future studies are warranted to examine if age may modify the expression of pain genes in older adults.

Additionally, genetic variations may influence the analgesic requirement or treatment responses to opioid analgesics [164]. A recent meta-analysis underscores that SNP A118G (a genetic variant of μ-opioid receptors, OPRM1) can modify postoperative opioid requirement and analgesic responses [165]. Notably, while Asians with minor G allele require more postoperative opioid analgesics, Caucasian counterparts do not display increased opioid analgesic requirements. This discrepancy highlights the genetic differences between the two ethnic groups and/or distinct interactions between A118G SNP and environmental influences [165]. Interestingly, the OPRM1 A118G SNP has significant influence only on the treatment responses of patients receiving morphine but not fentanyl [165]. The divergent pharmacogenetic responses indicate that different opioids may have different ligand-receptor dynamics [166]. Importantly, the expression of other pain genes (e.g., COMT or beta-2 adrenergic receptor alleles) [158, 167, 168] and other polymorphisms in the OPRM1 gene locus [169] can interact with A118G SNP and environment to cause differential pain sensitivity and opioid treatment responses in different races and gender [164, 170]. As such, it highlights that individual treatment responses of patients with LBP may be related to different pharmacogenetic variations.

#### Prior work exposures

While occupational exposures to whole-body vibrations, lifting, bending, twisting, stooping, have been identified as potential risk factors for LBP in the working-age group [171], increasing evidence suggests that previous occupational exposure to physically strenuous work increases the risks of LBP in retired seniors [172, 173]. A prospective study involving more than 1500 individuals showed that previous occupational biomechanical exposure to bending/twisting or driving for at least 10 years increased the odds of having persistent LBP in retired adults aged 58 to 67 years after adjusting for body mass index and psychological disorders [172]. Likewise, retired post office workers aged 70 to 75 years with LBP were characterized by more than 20 years of work-related regular lifting of heavy weights [173].

#### Demographic factors

Lower education levels, lower income, and smoking are related to higher propensity of LBP in older people [20, 21, 31, 112]. It is suggested that more educated individuals experience less LBP symptoms because they have a better understanding of pain, a better compliance to treatment, and a strong willingness to adopt a healthy lifestyle [174]. Conversely, people with poor economic status may have difficulty in accessing healthcare in certain places [175]. Patients with limited resources may delay seeking healthcare until their symptoms are intolerable, which in turn increases the chronicity/severity of LBP across the life course [176]. A multinational study has shown that people in the poorest socioeconomic quintile were 1.4 times more likely to have LBP with reference to the highest quintile [31]. Interestingly, compared to those older adults who have never married, those divorced, married, separated, and widowed have at least 1.5 times odds to experience LBP [31].

### Modifiable risk factors

#### Yellow flags

Psychological distress (e.g., anxiety or depression) is a risk factor for persistent or debilitating LBP in older adults [34]. A longitudinal study showed that older persons with a high depressive symptom score at baseline were two times more likely to have LBP at the 4-year follow-up [17]. Similarly, Reid et al. [177] found that depression was significantly correlated to disabling LBP in seniors aged 70 years or above. Importantly, since persistent LBP can also be a predictor of depression and anxiety [178], psychological assessments should be incorporated in the examination of older patients with chronic LBP.

Multiple studies have found that fear-avoidance beliefs (FAB) are closely related to chronic LBP in older people [179–181]. A cross-sectional study consisting of 103 older patients with chronic LBP (65 years or older) and 59-age-matched asymptomatic controls showed that higher FAB as measured by a questionnaire, older age, and higher LBP intensity predicted poorer self-reported functional capacity [179]. Another study on 200 older adults with chronic LBP revealed that higher physical activity subscale scores of the FAB questionnaire were related to higher Roland Morris Disability Questionnaire scores and slower gait speed [180]. Similarly, a population-based survey study found that increased FAB were related to higher self-reported LBP-related disability, poorer physical health, and higher risk of falls in older people (62 years or older) with LBP [181]. Vincent et al. also found that kinesiophobia was related to chronic LBP-related disability in obese older adults [182]. These consistent findings suggest that FAB are important therapeutic target to address among older people with chronic LBP.

Conversely, some studies reported inconsistent findings regarding the relation between other yellow flags (e.g., kinesiophobia and pain catastrophizing) and functional capacity or LBP-related disability [182–184]. A recent randomized controlled trial among 49 obese, older adults with chronic LBP demonstrated that reduction in pain catastrophizing following 4-month resistance exercise was related to decreased self-reported LBP-related disability [184]. However, Ledoux and coworkers found that kinesiophobia, pain catastrophizing, and depression were unrelated to the functional capacity among older adults with chronic LBP [185]. Kovac and colleagues also found that FAB and pain catastrophizing had only a minimal clinically significant effect on self-reported LBP-related disability of community-dwelling older (above 60 years) adults with LBP [183]. This discrepancy may be attributed to differences in study designs, cultures, living environment, or age-related changes in the relative influence of FAB on LBP-related disability level [183]. Given that multiple psychological factors (e.g., anxiety, depression, FAB, and coping strategy) may have different interactions among themselves and other age-related physical and social factors in influencing the genesis and persistence of chronic LBP, future studies should clarify the effect of individual yellow flags on LBP progression among older adults. The findings may help develop optimal multimodal treatment approaches for older adults with LBP [186].

#### Physical activity

Different types and amounts of physical activity are related to persistent LBP in older adults [112]. Generally, moderate or vigorous physical activity heightens the risk of LBP regardless of age [112, 171]. A population-based study found that moderate (at least 30 min of moderate intensity activity on five or more days per week) and vigorous (at least 20 min of vigorous activity on three or more days per week) physical activity were significantly associated with increased risk of persistent LBP among women aged greater than or equal to 65 years, while walking for 30 min on five or more days a week and strength exercises on two or more days per week lowered the risk of persistent LBP after adjusting for age and body mass index (BMI) [112]. Similarly, the study identified that strength exercises lowered the risk of LBP among men aged greater than or equal to 65 years after accounting for age and BMI [112]. As such, clinicians should evaluate the activity level of patients and provide recommendations accordingly.

#### Smoking

Like in other age groups, smokers are more likely to experience LBP. It is thought that smokers may have different pain perception as compared to non-smoker although the effect of smoking on pain perception remains unclear [187]. However, animal and human studies have shown that smoking may induce degenerative changes in spinal structures, such as intervertebral discs [188–191]. As such, these degenerative changes may compress the neural structures and cause neuropathic LBP.

#### Social factors

Social factors may affect the genesis and persistence of LBP [192]. It is well known that social factors (e.g., the social environment or groups that individuals live, grow up, or belong) can influence the onset and progression of diseases or disability (including widespread pain) [193, 194], especially among older adults [195, 196]. Because social conditions can induce social stressors (e.g., poor housing, crime, and poor living environment), affect risk exposure (e.g., poor eating habit leading to obesity), influence psychology and emotion (e.g., social pressure and sense of inequalities), and compromise access to health services (e.g., health-care education or use of healthcare) [192]. Health-care stakeholders should recognize and address various social factors that can impact older adults with LBP. For example, since older adults with less social ties are more likely to experience disabling pain because of depression [192], proper public health programs and resource allocation (e.g., social work counseling services and health education) may target these vulnerable seniors (e.g., oldest old or seniors with depression). Importantly, residents with LBP living in long-term care facilities may rely on nursing home staff (e.g., nursing assistants) to provide medications or personal care. The attentiveness and responsiveness of nursing home staff will affect the recovery and persistency of LBP in these residents.

#### Self-perceived health

Seniors with poor self-perceived health status are more likely to experience severe LBP. A cross-sectional study on adults aged between 70 to 102 years found that poor self-rated health was strongly associated with LBP [197]. Similarly, a longitudinal study revealed that people with poor self-reported health were four times more likely to report LBP at the 4-year follow-up than those reporting very good health [17]. The same study also found that those who required health or social services (e.g., meals on wheels or home help) at baseline had a significantly higher risk of reporting LBP at follow-up [17].

#### Comorbidity

Research has shown that comorbidities are related to chronic LBP in seniors. Jacobs et al. [35] found that females, hypertension, joint pain, pre-existing LBP, and loneliness, were predictors for developing persistent LBP in individuals aged 70 years. Another study revealed that comorbid chronic conditions were positively related to at least one LBP episode in the last month in low- and middle-income countries [31]. Specifically, the odds of LBP were 2.7 times higher among seniors with one chronic comorbid condition, compared to seniors without comorbidities, while the odds ratio was 4.8 for people with two or more comorbidities [31]. As mentioned above, patients with Parkinson’s disease may experience hypersensitivity of pain due to the decrease in striatal dopaminergic function [198, 199]. However, such pain can be alleviated by the administration of L-dopa [200].

### Special considerations for low back pain management of seniors

While comprehensive history taking, self-reports of pain characteristics and pain-related disability, as well as proper physical examination all are necessary for differential diagnosis among older adults with LBP [201], attention should be also given to assessment and treatment of seniors with LBP so as to optimize pain management (Fig. 1).

#### Self-reported pain assessments

While patients with mild-to-moderate dementia can reliably report pain intensity using traditional visual analog scale or Numeric Rating Scale (NRS) [202, 203], other self-reported pain assessment tools have been developed and validated in the older population to improve pain evaluation (Table 1). The 11-point NRS is commonly used in clinical settings, where 0 means no pain and 10 means the worst pain imaginable [204]. Faces Pain Scale and Revised Faces Pain Scale (FPS) comprise different facial expressions indicating different severity of pain experienced by patients [205]. They have been validated among different older populations [168, 204, 206–208] and were rated as preferred tools over the NRS by Chinese [209] and African-Americans [210]. The Iowa Pain Thermometer (IPT) is a descriptor scale presented alongside a thermometer to help patients conceptualize pain intensity as temperature levels [204]. Compared to the FPS, Verbal Descriptor Scale, and visual analog scale, the IPT is deemed to be the most preferred scale among older adults [204].

**Table 1:** Self-reported pain assessment tools for older adults with cognitive impairment

Scale	Description	Psychometric properties
Numeric Rating Scale (NRS) [204]	A line with numbers 0 to 10 displayed at equal intervals, where 0 means no pain and 10 means the worst pain imaginable.	NRS has been validated among older adults [300, 311, 312]. The completion rate was high for people with cognitive impairment. The completion rate decreased in people with mild (76%) to moderate (58%) cognitive impairment [313].
Faces Pain Scale (FPS) Revised Faces Pain Scale (FPS-R) [206, 207]	Consists of different facial expressions to indicate different severity of pain experienced by patients.	Both are reliable and valid in older people with cognitive impairments and with different cultural background [204, 209, 210, 314, 315] For patients with deficits in facial recognition, the results should be interpreted with care [316].
Iowa Pain Thermometer (IPT) [204]	A descriptor scale presented with a graphic thermometer showing a color gradient from white to red in order to help patients rate their pain intensity as temperature. Additional choices between words are available to improve the sensitivity of the scale.	Older adults with cognitive impairment are more likely to correctly complete IPT as compared to NRS, Verbal Descriptor Scale, FPS, and visual analog scale [204]. IPT is the most preferred scale by both young and older adults (with osteoarthritic pain) [204].
Verbal Descriptor Scale (VDS) [317]	Consists of seven verbal descriptions to indicate different severity of pain ranging from 0 to 6, where 0 means “no pain” and 6 means “pain as bad as it could be.”	VDS score agrees with the ratings of FPS or NRS but their associations are not linearly related [317]. The majority (90%) of people with moderate cognitive impairment can accurately use VDS [313]. A simplified version has been developed for people with severe dementia [318]
Visual Analog Scale (VAS) [319]	A 10-cm line with 0 means no pain and 10 means the worst possible pain.	VAS has significantly higher error (approximately 20%) among older adults as compared to NRS and VDS [203, 320, 321].

#### Observational pain assessments

Although self-reported pain assessment is the gold standard, clinicians need to validate the self-reported pain with observed pain behavior during physical examination. While some seniors with cognitive impairment may report exaggerated pain without coherent pain behavior due to perseveration [211–214], others (e.g., with severe dementia or poststroke aphasia) may have difficulty in communicating pain intensity or pain-related disability [215] that may lead to insufficient/inappropriate treatment [216]. Currently, there is no consented guideline regarding the relation between the trustworthiness of self-reported pain and cognitive functioning [217]. Therefore, health-care providers (e.g., physicians or nursing home nurses) should identify people with potential cognitive impairment and modify their pain assessment and treatment in order to effectively manage cognitively impaired patients with LBP. It has been suggested that clinicians should consider assessing the cognitive function of older adults with LBP if patients have a known history of dementia, self or family report of memory loss, difficulty in providing details of LBP history that requires supplementary input from caregivers, age above 85 years, or inconsistency between observed pain behaviors and self-reported pain [212, 213, 218]. Some dementia screening tools (e.g., Montreal Cognitive Assessment [219], Mini-Cog [220, 221], and Saint Louis University mental status examination [222]) have been recommended based on their psychometric properties, ease of use, and accuracy in identifying people with dementia [223]. Patients with positive screening results should be referred to subspecialty dementia experts (e.g., neurologists, geriatricians, or geriatric psychiatrists) for formal dementia evaluation in addition to LBP treatment. Collectively, early identification of cognitive impairment and psychiatric comorbidity (e.g., depression) in older adults with LBP can optimize the pain management plan (e.g., assistance from caregivers and prescription of psychiatric medications).

Since people with moderate to severe dementia may display agitation, anxiety, or nonverbal pain behaviors (e.g., grimacing, yelling, hitting, or bracing), failure to detect pain as a potential cause of agitation may result in unnecessary prescription of anxiolytics or antipsychotics [224]. As such, proper procedures for evaluating nonverbal dementia patients should include: using a validated observational assessment tool to evaluate pain behaviors during rest and painful conditions/procedures, seeking surrogate report of pain behaviors, and monitoring responses following an analgesic trial [223]. Since the prevalence of dementia in people aged 85 or older can be as high as 50% [218], family members or informants are recommended to accompany these patients to meet health-care providers so as to provide detailed pain information [223]. Several recent reviews have identified at least 24 observational pain assessment instruments for estimating pain in nonverbal patients [225–227]. Table 2 describes six commonly used assessment instruments. Unfortunately, since many of them only detect the presence/absence of pain, rather than quantify the pain severity [217, 228], these tools may be better used to monitor longitudinal changes in pain (e.g., increases/decreases in pain behavior) or treatment responses. Regardless, if the observational pain behavior assessment indicates the presence of significant pain in patients, the sources of pain should be identified through physical examination and proper treatment should be given. If inconsistency occurs between the observational assessment and self-report of pain, other causes (e.g., fear of pain and depression) should be identified and managed. If comprehensive evaluations and an analgesic trial cannot identify any sources of pain experienced by patients with dementia, the persistent pain complaint may be attributed to pain perseveration, which is the repetitive reporting of pain without actual distress. Collectively, future studies should refine existing observational tools by identifying the most important behaviors for evaluating the presence and severity of pain (including LBP) in cognitively impaired patients.

**Table 2:** Six commonly used nonverbal pain tools for older adults with cognitive impairment

Scale	Description	Psychometric Properties
Checklist of Nonverbal Pain Indicators (CNPI) [322]	An observational scale monitoring pain behaviors in 6 behavioral items (vocal complaints, nonverbal sound, facial grimace/winces, bracing, rubbing, and restlessness) at rest and during movement. An item is rated 0 or 1 based on the absence or presence of a pain behavior. The presence of any of the pain behavior indicates pain. There are no cutoff scores to represent pain severity.	Nursing home residents. Good internal consistency (*α* = 0.92 to 0.97 at rest; *α* = 0.74 to 0.90 during movement); Good construct validity but having a great floor effect at rest [323] Good construct validity against NOPPAIN, PACSLAC, and PAINAD (*r* = 0.66 to 0.71) [324] Interrater reliability for behaviors (*K* ranged from 0.63 to 0.82) [322] moderate inter-rater reliability at rest (K = 0.43); Fair inter-rater reliability with movement (*K* = 0.25) [323] Test-retest reliability ranged from 0.44 to 0.56, inter-rater reliability ranged from 0.58 to 0.71, internal consistency *α* ranged from 0.76 to 0.82, and factor analysis revealed that CNPI might have more than 1 factor [325]. Older patients with hip fracture in a surgical ward internal consistency (*α* = 0.54 at rest; *α* = 0.64 with movement) [322]
The Abbey Pain Scale (APS) [326]	For people with end-stage dementia. Comprises 6 questions regarding the facial expression, vocalization, change in body language, behavioral change, physiological change, and physical changes. Each question can be given a score from 0 to 3, where 0 means absence while 3 means severe. Higher total scores indicate higher pain intensity.	The Australian Pain Society has endorsed this scale for evaluating pain in older people with dementia [226]. Nursing home residents. A strong agreement (66.1 to 78.3%) between proxy-reported APS scores and presence of self-reported pain, moderate correlation between self-reported pain intensity and APS (*r* = 0.56; *p* < 0.01) residents with cognitive impairment; 25.4% above chance to correctly identify cognitively impaired patients with pain [300]. Concurrent validity between the APS and nurse’s holistic pain assessment was acceptable (Gamma = 0.59; *p* < 0.01), internal reliability (*α* = 0.74–0.81), inter-rater reliability was modest (but no actual statistics) [326] Moderate to good construct validity against PACSLAC and PAINAD at rest and exercise (*r* = 0.56–0.85) [327] Test-retest reliability (*r* = 0.62–0.68), inter-rater reliability (ICC = 0.70–0.75), internal consistency (*α* = 0.65*–*0.80). Factor analysis only revealed 1 single factor [325]. Patients in Geriatric wards It has been translated into Danish and tested on severely demented and non-communicative older patients in geriatric wards. There was a poor agreement between APS and verbal rating scale (*k* = 0.42), interrater reliability was good (ICC = 0.84). Fair internal consistence (Cronbach’s *α* = 0.52) [328].
The Doloplus 2 [329]	10-item scale evaluating three domains: (1) somatic, (2) psychomotor, and (3) psychosocial; Each item has four potential scores, where 0 means normal behavior and 3 indicates high levels of pain-related behavior. It is administered by a trained nurse.	It was originally developed in French but has been translated into English. Two systematic reviews rated Doloplus 2 as a scale with high-psychometric properties [226]. Nursing home residents. Internal consistency (*α* = 0.82–0.87) [325, 330]. Criterion validity between Doloplus-2 score rated by a geriatric expert nurse and pain evaluation conducted by a pain expert (R2 = 0.54); inter-rater reliability (ICC = 0.74–0.77). Small but significant correlation between the expert’s pain in movement score and the Doloplus-2 item for protective body at rest score and for the expert’s pain at rest score (R2 = 0.12; *p* < 0.01) and between Doloplus-2 item and pain complaints (R2 = 0.13; *p* < 0.01) [330]. Factor analysis only revealed one single factor [325]. Test-retest reliability (*r* = 0.71), inter-rater reliability (ICC = 0.73–0.81) [325].
Noncommunicative Patient’s Pain Assessment Instrument (NOPPAIN) [331]	A nursing assistant-administered observation tool for recognizing and rating of extent of pain behaviors. Contains four sections considering six pain behaviors (pain-related words, facial expression, pain noises, rubbing, bracing, and restlessness) during common care conditions (e.g., bathing). Each pain response can be rated from 0 to 5 on a surrogate Likert scale, where 0 indicates the lowest possible intensity and 5 means the highest possible intensity.	The National Nursing Home Pain Collaborative acknowledged the scale in evaluating pain behaviors but reported that the complexity of NOPPAIN might limit its clinical use [225]. It has to be validated in clinical setting. Nursing home setting. Excellent agreement (*k* = 0.87) for assessing video tape results [300]. Strong agreement (69.2 to 80.0%) between proxy-rated pain behaviors and self-reported presence of pain [300]. Moderate correlation between self-reported pain intensity and NOPPAIN (*r* = 0.68; *p* < 0.01) in residents with cognitive impairment. There was 25.4% above chance to correctly identify cognitively impaired patients with pain [300]. Good construct validity against CNPI, PACSLAC, and PAINAD (*r* = 0.71–0.78) [324, 327]. High intra-rater reliability (*k* = 0.70–0.86), high inter-rater reliability (*k* = 0.72*–*1.0) [324, 331, 332].
Pain Assessment Checklist for Seniors with Limited Ability to Communicate (PACSLAC) [333]	PACSLAC evaluates 60 pain behaviors classified into four subscales: (1) facial expression, (2) social behavior mood and personality, (3) physical activity and body movement, and (4) physiological changes, eating or sleeping changes, and vocal behaviors. PASCLAC-II consists of 31 items by removing items that may be mixed with signs of delirium [334].	Both PACSLAC and PACSLAC-II cover all observational pain assessment domains recommended by the American Geriatrics Society Guideline [301, 335]. Two systematic reviews also suggest PACSLAC as one of the psychometrically strongest assessment tools [226, 227]. Nursing home settings. PACSLAC internal consistency (*α* = 0.62–0.92) [336, 337]. PACSLAC-II internal consistency (*α* = 0.74–0.77) [334]. Moderate correlations between PACSLAC scores and global pain intensity ratings (*r* = 0.39–0.54) [337]. Good correlation between PACSLAC scores and VDS (*r* = 0.81) and VAS (0.72–0.86) for unblended rating of acute influenza injection [336]. Both PACSLAC and PACSLAC-II have demonstrated good differentiation between painful and non-painful states in patients (*p* < 0.01) [324, 334]. Good construct validity against NOPPAIN and PAINAD (*r* = 0.66–0.78) [324]. Good construct validity against APS (*r* = 0.79) [327]. PACSLAC and PACSLAC-II have strong correlation (*r* = 0.81–0.89) and the NOPPAIN (*r* = 0.73) [334]. Inter-rater reliability at rest and during movement (ICC ≥0.76) [299, 324, 327]. Inter-rater reliability (*k* = 0.63) for PACSLAC-II [334]. Excellent inter-rater reliability (ICC = 0.93–0.96); Intra-rater reliability (ICC = 0.86) for unblended rating of acute influenza vaccination [336].
The Pain Assessment in Advanced Dementia (PAINAD) Scale [228, 338]	A 5-min observation during activity. It evaluates five behaviors (breathing, negative vocalization, facial expression, body language, and consolability) as five indicators of discomfort rated on three levels: 0=absent, 1=present but not constant or severe, 2=severe/constant.	The National Nursing Home Pain Collaborative recommended the PAINAD for clinical use [225]. It has been validated in acute care setting and nursing homes [339]. Nursing home settings. High internal consistency (*α* > 0.70) [323, 327]. It can detect the presence or absence of pain but not the severity of pain [340]. Strong agreement (66.1 to 73.3%) between PAINAD and proxy-rated pain behaviors or self-reported presence/absence. There was 19.2% above chance to correctly identify cognitively impaired patients with pain [300]. High correlation between PAINAD scores and nurses’ pain reports (Kendall’s *τ* = 0.84) [341]. PAINAD scores decreased following administration of analgesics and changes with potentially painful activity [324, 336]. Good construct validity with CNPI, APS, NOPPAIN, and PACSLAC at rest and during exercise (*r* = 0.56–0.90) [324, 327] High inter-rater reliability (*r* = 0.80–0.97) and test-retest reliability (*r* = 0.90) [228, 342, 343].

It is noteworthy that although certain physiological parameters (e.g., increased heart rate, blood pressure, and perspiration) may indicate the presence of pain, these physiological indicators may be inaccurate among older adults with chronic pain [217]. Additionally, older adults with dementia may have diminished autonomic reactions to pain [229, 230]. Therefore, effective evaluation of pain behavior may be more relevant for older adults with severe dementia and pain.

#### Fall assessment and prevention

Given that older people usually display reduced physical capacity [231], cardiac output [232], muscle mass and strength [233], and older adults with LBP are more likely to suffer from decreased mobility and functional deterioration than younger sufferers. In addition, older adults with musculoskeletal pain are more likely to experience fear of falling [234] and fall incidents [23]. Specifically, LBP is known to be an independent risk factor for repeated falls in older women [235]. A prospective study revealed that community-dwelling seniors with chronic LBP (more than 3 months) had a significantly higher risk of falls (adjusted OR for injurious falls ranged from 2.11 to 2.46) as compared to asymptomatic counterparts [236]. Likewise, seniors with LBP in the past 12 months are more likely to be recurrent fallers [23]. Since falls is the leading cause of persistent pain, disability, and mortality among seniors [36, 237], physicians and nursing home workers should assess fall risks of older adults with LBP [238] and refer them for fall prevention intervention, if necessary.

#### Pain medications

The American Geriatrics Society has published recommendations on pain management of geriatric patients with nonmalignant pain. In particular, a standing order of analgesic (e.g., acetaminophen) is recommended for older adults with chronic pain so that they can have a steady concentration of analgesic in the blood stream [239]. Tramadol is recommended to be prescribed with caution for patients with a known risk of seizure (e.g., stroke, epilepsy, and head injury) or for those taking medications that may lower seizure threshold (e.g., neuroleptics and tricyclics) [239]. In addition, the guideline also suggests that if acetaminophen cannot control pain, non-steroidal anti-inflammatory drugs (NSAIDs) (e.g., COX-2 therapy or non-acetylated salicylates) may be used as adjunct therapy [239]. However, since some traditional NSAIDs may cause gastrointestinal upset, clinicians are recommended to prescribe non-acetylated salicylates for older patients with peptic ulcer and gastrointestinal bleeding. Although there is no ideal dose for opioid prescription among older adults with LBP, the effective dose should be carefully titrated to fit individual needs. To attain better pain relief with minimal side effects secondary to a high dose of a single medication, it is recommended to concurrently use two or more pain medications with different mechanisms of action or different drug classes (e.g., opioid and non-opioid analgesics). It is noteworthy that opioid (e.g., codeine) may increase the risk of falls and other drug-related adverse effects (e.g., depression, nausea, tachycardia, seizure, or falls [240, 241]) in opioid-naïve older patients during the opioid initiation period (i.e., within the first 3 months) or during the use of long-acting opioids [242, 243]. Therefore, specific education and caution should be given to these patient groups.

In addition, because older patients with chronic LBP are commonly associated with depression or anxiety, it is not uncommon for them to take antidepressants (e.g., serotonin reuptake inhibitors) or benzodiazepines. Since some of these psychoactive drugs may compromise their memory, cognition, alertness and motor coordination [244, 245], special care should be given to these patients to minimize their risks of falls, hip fractures, or road traffic accidents [246]. For instance, concurrent prescription of tramadol and the selective serotonin reuptake inhibitor (an antidepressant) may increase the risk of serotonin syndrome (e.g., hyperthermia, agitation, diarrhea, tachycardia, and coma) that may lead to sudden death [247, 248]. If patients have an elevated risk of opioid overdose (e.g., alcoholism [249], a history of opioid overdose/drug abuse [250], concurrent consumption of benzodiazepine or sedative hypnotics [251], or poor compliance to opiate medications [252]), they should undergo an overdose risk assessment, a urine drug abuse screening prior to opioid prescription, an education on drug overdose, and frequent clinical follow-up so as to mitigate their risk [253]. Further, physicians can prescribe naloxone to these high-risk patients and teach them/their caregivers to use it at emergency. Naloxone is an opiate antidote for neutralizing the toxicity of opioid overdoses [253, 254]. For patients who are taking long-acting opioids (e.g., oxycodone or methadone) or having hepatic or renal dysfunction, they should be reassessed regularly in order to ensure timely tapering/discontinuing of opioids if necessary [253]. Collectively, existing medical guidelines generally recommend low-dose initiation and gradual titration of opioid therapy and constipation prophylaxis, increased awareness of potential interactions among concurrent medications, as well as close monitoring of treatment responses in patients. It is necessary to provide updated education to health-care providers so as to optimize pain management for older patients with chronic pain.

#### Other conservative treatments

Although analgesics are the first line treatment for older people with LBP, older people with LBP (especially those with a prolonged history of LBP) may require other conservative treatments to mitigate pain and to restore function. Growing evidence has indicated that some, but not all, conservative treatments can benefit older people with LBP [255, 256]. While the efficacy of various physiotherapy modalities in treating older people with LBP remains controversial [256], a recent meta-analysis has highlighted that Tai Chi, a mind-body exercise therapy, is an effective intervention for older patients with chronic pain (including LBP, osteoarthritis, fibromyalgia, and osteoporotic pain) as compared to education or stretching [255]. Importantly, in addition to pain relief, various systematic reviews on Tai Chi have revealed promising outcomes in improving balance [257], fear of falling [258], lower limb strength [259], physical function [260], hypertension [261], cognitive performance [262], and depression [263] in seniors as compared to no treatment or usual care. Given the high frequency of physical and psychological comorbidity among older adults (e.g., depression, hypertension, and osteoarthritis), Tai Chi appears to be a viable LBP treatment option for older adults with LBP. Future studies should determine the dose response of Tai Chi in treating older people with LBP in community and institutional settings.

#### Lumbar surgery

Surgical intervention is indicated for older people only if there is a definite diagnosis of lumbar pathology (e.g., degenerative LSS, cauda equine syndrome, or spinal tumor) that needs to be treated by surgery or that is unresponsive to conservative intervention. While there are many different lumbar surgical interventions, the objective of these approaches is to minimize compression of neural tissues and/or enhance spinal stability. Decompression surgery (i.e., laminectomy, laminotomy, and discectomy) is used to partially or completely remove lumbar structures that are impinging neural tissues [264, 265]. Recent evidence suggests that minimally invasive spine surgery techniques have higher success rate than open lumbar decompression surgery [266]. Unlike decompression surgery, spinal fusion surgery utilizes bone grafts (autograft or allograft) or surgical devices to fuse adjacent vertebrae anteriorly, posteriorly, or circumferentially. Such surgery immobilizes the spinal motion segment, in theory removes key pain generating sources and eliminates intersegmental movement of vertebrae that may compress neural structures in order to alleviate symptoms [267]. In general, both simple and complex spinal fusion surgeries are associated with a higher risk of major complications and postoperative mortality as compared to decompression surgery [264]. While decompressive laminectomy/laminotomy with or without spinal fusion is a common surgical intervention for older patients with degenerative LSS [268], isolated decompression without spinal fusion is a preferred choice for older patients with lumbar degenerative spondylolisthesis without severe LBP/instability [269]. However, two recent randomized controlled trials have reported conflicting results regarding the effectiveness of decompression surgery plus spinal fusion versus decompression surgery alone in treating patients with LSS and degenerative spondylolisthesis [270, 271]. Decompression and spinal fusion are also indicated for patients with symptomatic degenerative lumbar scoliosis [272, 273] although these procedures may increase the risk of complications in older adults (especially those with comorbidities) [268, 272, 274–276]. Recently, disc arthroplasty has been adopted to restore the mobility of an intervertebral joint by replacing a degenerative disc with an artificial disc and minimizing the risk of adjacent segment degeneration/disease [277]. Although current evidence notes the safety and efficacy of such intervention for indication for cervical spine pathology in comparison to conventional interbody fusion procedures, outcomes for lumbar disc disorders remain under further evaluation.

Percutaneous transpedicular vertebroplasty and balloon kyphoplasty are two minimally invasive techniques for treating patients with painful osteoporotic vertebral compression fracture [278]. These procedures involve the injection of a small amount of bone cement into the collapsed vertebral body to alleviate excruciating pain and stabilize the fractured vertebral body [279]. However, individual studies have found that these procedures may heighten the risk of new vertebral fractures at the treated or adjacent vertebrae, and other complications (e.g., cement leakage into the lungs, veins, and the vertebral body) [280–283]. However, a recent meta-analysis reveals that these vertebral augmentation procedures may attenuate pain and correct deformity of patients with osteoporotic vertebral compression fractures without increasing the risk of complications or new vertebral fractures along the spine [278].

In addition, the past decade alone has seen a significant interest in the concept of sagittal alignment and balance with respect to the preoperative planning and predictive outcome analyses of patients with various lumbar spinal disorders and spinal deformities [284, 285]. Novel imaging software has been developed to quantify such parameters, such as pelvic incidence and tilt, and sacral slope, in a semi-automatic fashion [286, 287]. Numerous studies have noted the clinical utility assessing spinal alignment/balance [288–292] a field that continues to gain widespread momentum and motivate future research.

Like conservative LBP treatments, some patients may experience persistent LBP (with or without sciatica) even after spinal surgery. The reasons for the failed back surgery syndrome (FBSS) may be ascribed to technical failure, incorrect selection of surgical patients, surgical complications, or related sequelae [267]. Additionally, since spinal surgery may alter the load distribution at vertebral structures adjacent to the operated segments (e.g., sacroiliac joint), this may result in the adjacent segment disease and pain. Because patients with FBSS are unlikely to benefit from revision surgery, spinal cord stimulation has been suggested to manage pain in these patients. Specifically, spinal cord stimulation involves the placement of electrodes into the epidural space and the generation of electrical current by a pulse generator placed subcutaneously. Studies have noted that there is fair evidence to support moderate effectiveness of spinal cord stimulation in attenuating persistent radicular pain of appropriately selected patients with FBSS although device-related complications are also common [267].

It is noteworthy that while surgical intervention may benefit some patients with LBP, clinicians should weigh the risks and benefits of surgery for each individual patient. A recent Cochrane review summarized the evidence regarding the effectiveness of surgical and conservative treatments for patients with LSS [293]. Two of the five included randomized controlled trials reported that patients undergoing spinal decompression with or without fusion had no significant difference in pain-related disability (measured by Oswestry Disability Index) from those receiving multi-modal conservative care at 6 and 12 months although the decompression group demonstrated improved disability at 24 months [294, 295]. Similarly, a small-scale included study found no significant difference in pain outcomes between decompression and usual non-surgical care (bracing and exercise) at 3 months, and 4- and 10-year follow-ups [296]. Another included study revealed that minimally invasive mild decompression was no better than epidural steroid injections in improving Oswestry Disability Index scores at 6 weeks although decompression had significantly better pain reduction but less improvement in Zurich Claudication Questionnaire scores [297]. Conversely, an included trial found that an interspinous spacer was significantly better than usual non-operative care in reducing symptoms and restoring physical function at 6 weeks, and 6 and 12 months [298]. Regardless of the treatment effects, approximately 10 to 24% of participants experienced peri or postoperative complications (e.g., lesion to the dural sac, hematoma, infection, spinous process fracture, respiratory distress, coronary ischemia, stroke, and even death secondary to pulmonary edema) while no side effect was documented for any conservative treatments [293]. Given above, back surgery should be considered carefully for high-risk patients (e.g., older adults with medical comorbidity). High-quality randomized controlled trials are warranted to compare the effectiveness of surgical versus nonsurgical interventions for older patients with LSS.

### Future research

While anecdotal evidence and clinical experience suggest that older people appear to have higher rates of LBP with definite pathology (e.g., vertebral osteomyelitis, degenerative spondylolisthesis, and DNDLS), only a few studies have properly evaluated this issue. Given this knowledge gap, future research should quantify the prevalence of various LBP diagnoses so that health care resources can be better allocated to effectively manage the epidemic of LBP in the older population.

Although self-report of LBP is the gold standard for evaluating subjective pain experience, some patients with cognitive impairment may be unable to effectively verbalize their pain. Clinicians (especially those working in the geriatric field) should improve their competence in assessing nonverbal pain expression in patients with cognitive impairment. While multiple observational pain assessment scales have been developed, there is no consensus on the use of a particular assessment tool. Different clinical guidelines have recommended different scales [223, 225]. Given the rapid development and validation of different observational scales in the last decade, it is necessary to update existing guidelines on this issue.

While the scores of several observational pain behavior assessment tools (e.g., the Abbey Pain Scale and Pain Assessment in Advanced Dementia) have been found to be closely related to self-report of pain [299, 300], there is a paucity of research on the interpretation of scale/subscale scores in relation to pain or other psychological comorbidity (e.g., depression). Future studies should establish this relation. Further, most of the existing behavioral observational pain scales have only been validated in the nursing home setting. Future studies are warranted to compare various existing scales and evaluate their responsiveness and sensitivity to changes in pain following treatments in different settings, which can identify best assessment tools for different settings.

Since recent findings suggest that facial expression can provide many useful indirect information of pain, training health-care providers on the recognition and interpretation of facial expression of pain may improve the accuracy and reliability of pain assessment among patients with dementia. Importantly, future studies should adopt computer vision technology to develop automatic, real-time assessment of pain-related facial expression so as to facilitate the evaluation of pain condition in non-communicable patients with LBP [301].

Currently, clinical assessments of LBP among older adults rely heavily on self-report or surrogate report of LBP or manual physical assessments. With recent advances in technology, clinicians can use reliable novel objective measurements (e.g., mechanical spinal stiffness assessments [302–304], ultrasonic measurements of paraspinal muscles [305], advanced medical imaging [306, 307], or genetic analysis [308]) to examine patients at affordable costs. Given that age-related physical changes (e.g., sarcopenia or fatty infiltration of paraspinal muscles) in older adults may worsen LBP-related physical changes, the adoption of validated objective measurements may enhance the reliability and sensitivity in detecting physical deficits or monitoring posttreatment improvements of LBP in older adults. For example, ultrasonography may be used to quantify atrophy of lumbar multifidus that can guide clinical treatments (e.g., spinal stabilization exercises). Likewise, computerized spinal stiffness tests can be used to identify patients with LBP who are likely to benefit from spinal manipulation [309]. Novel yet more sensitive imaging, such as chemical exchange saturation transfer, T2 mapping, T1-rho, ultra-short time-to-echo and sodium MRI, may identify the pain-generating source allowing for more targeted therapies [50, 310]. Furthermore, a refinement of some of the imaging phenotypes (e.g., disc degeneration, endplate changes, facet joint changes, paraspinal muscle integrity, and sagittal alignment/balance) or the utility of “phenomics” may further aid in proper diagnosis, management options, and the potential development of novel therapeutics. Knowledge gained from such approaches may enhance the exploration of new pathways of pain and potential treatment options in appropriate animal models. Moreover, the role of pain genetics and its actual utility toward the management of LBP in older individuals needs to be further explored. Taken together, while novel technology may gather new information from patients with LBP, clinicians should integrate these objective outcomes with other clinical findings in order to make proper diagnosis and clinical decision.

Given the multifactorial causes of LBP in older adults, it is necessary to consider the entire spectrum of “omic” approaches (e.g., genomics, metabolomics, phenomics, etc), ethnic variations, and all aforementioned risk factors in order to derive appropriate predictive models for future LBP development or severity of pain. These models can then be used to develop cost-effective and personalized LBP intervention for older adults.

## Conclusions

Although LBP is ubiquitous among older adults, the dearth of literature on the trajectories of LBP, determinants of chronic LBP, and effective LBP managements in older adults highlights the research gaps in this area. Given that multiple factors (e.g., dementia, psychiatric and physical comorbidities, maladaptive coping, and age-related physical and psychosocial changes) can modify the LBP experience in older adults, clinicians should incorporate comprehensive subjective, observational, and physical examinations, as well as proxy reports to make accurate diagnosis. For patients with persistent LBP, medical imaging may be ordered to rule out malignant causes of pain. To minimize undertreatment of older adults with LBP, it is necessary to recognize the presence of LBP and to titrate pain medications in accordance with individual needs. Through understanding various factors contributing to severe/chronic LBP in older adults, timely and proper treatment strategies can be formulated. In addition, with the expansive understanding of “omic” technologies, study designs, and findings, new pathways of pain may be identified and novel therapeutics may be developed. As such, it is with a hope that with the understanding of pain being broadened and deepened, the management of older patients with LBP may eventually become more personalized or precise and outcomes optimized, leading to a healthier and productive society.

## Abbreviations

APS: Abbey Pain Scale
BMI: Body mass index
CNPI: Checklist of Nonverbal Pain Indicators
DNDLS: De novo degenerative lumbar scoliosis
FAB: Fear avoidance beliefs
FPS: Faces Pain Scale
IPT: Iowa Pain Thermometer
LBP: Low back pain
LSS: Lumbar spinal stenosis
MRI: Magnetic resonance imaging
NOPPAIN: Noncommunicative Patient’s Pain Assessment Instrument
NRS: Numeric Rating Scale
NSAID: Non-steroidal anti-inflammatory drug
PACSLAC: Pain Assessment Checklist for Seniors with Limited Ability to Communicate
PAINAD: Pain Assessment in Advanced Dementia Scale
SNP: Single-nucleotide polymorphism
VAS: Visual Analogue Scale
VDS: Verbal Descriptor Scale
VO: Vertebral osteomyelitis
